# Supplementary figures and images for: A natural mutation between SARS-CoV-2 and SARS-CoV determines neutralization by a cross-reactive antibody
Source: PLoS Pathog. 2020 Dec 4;16(12):e1009089. doi: 10.1371/journal.ppat.1009089 (PMC7744049; doi:10.1371/journal.ppat.1009089)

Figure S1

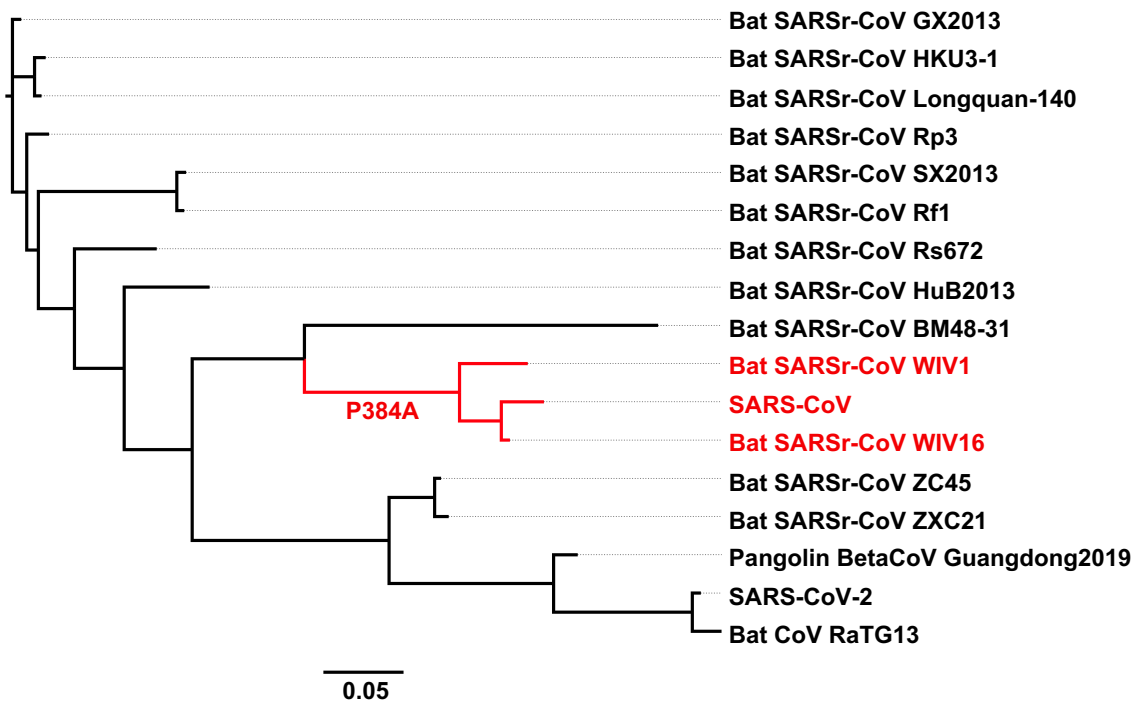

Supplement: S1 Fig — Branches corresponding to strains that have A384 are colored in red on the phylogenetic tree. Scale bar represents 0.05 amino-acid substitutions per position. (PDF) [file ppat.1009089.s001.pdf]

**Figure S2**

**A**

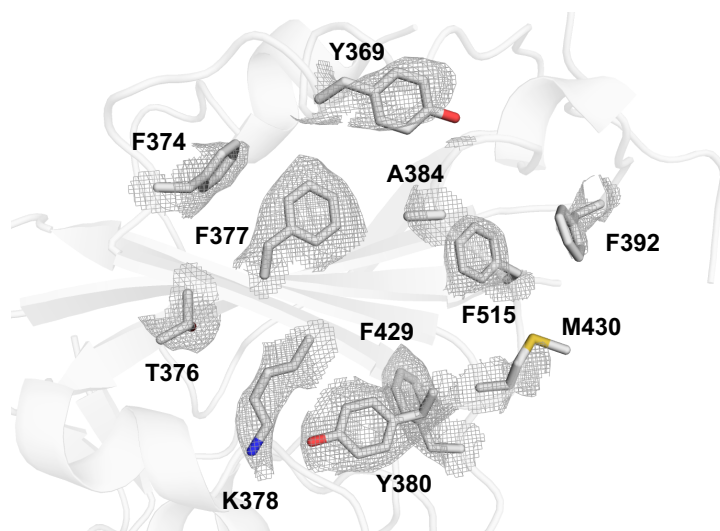

**B**

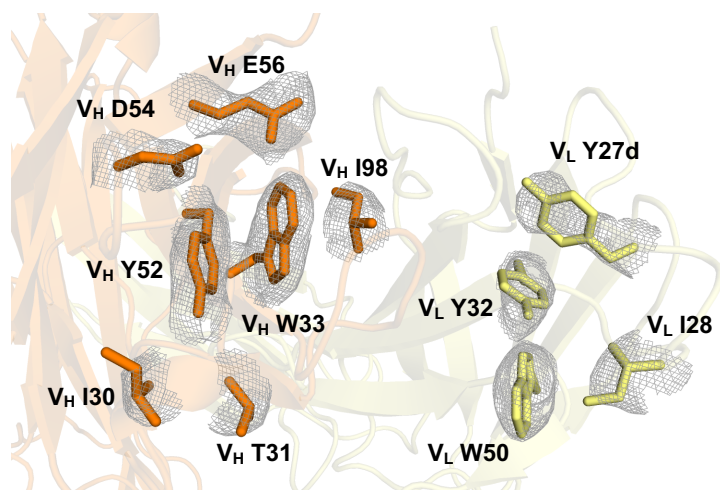

Supplement: S2 Fig — (A) Final 2Fo-Fc electron density maps for the side chains in the epitope region of SARS-CoV-2 contoured at 1 σ. (B) Final 2Fo-Fc electron density maps for the paratope region of CR3022 contoured at 1 σ. The heavy chain is colored in orange, and light chain in yellow. Epitope and paratope residues are labeled. (PDF) [file ppat.1009089.s002.pdf]

**Figure S3**

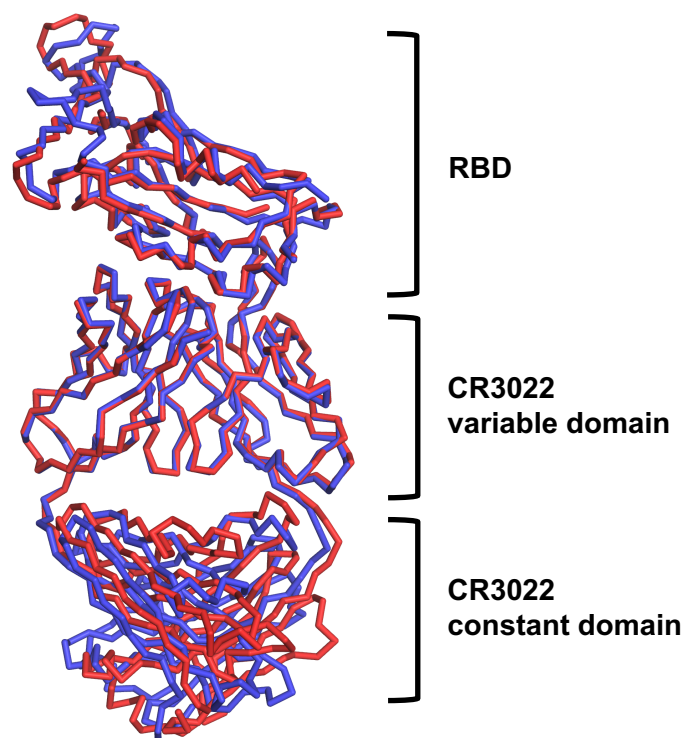

Supplement: S3 Fig — Structure of CR3022 in complex with SARS-CoV RBD (this study) is aligned to that with SARS-CoV-2 RBD (PDB 6W41). Structural alignment was performed using CR3022 heavy chain variable domain. Red: CR3022 in complex with SARS-CoV RBD. Blue: CR3022 in complex with SARS-CoV-2 RBD. (PDF) [file ppat.1009089.s003.pdf]

**Figure S4**

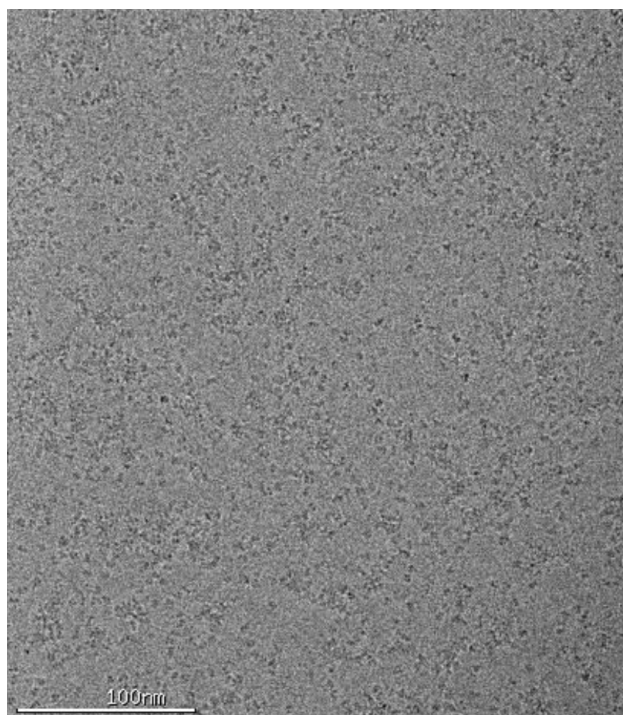

**2D class averages**

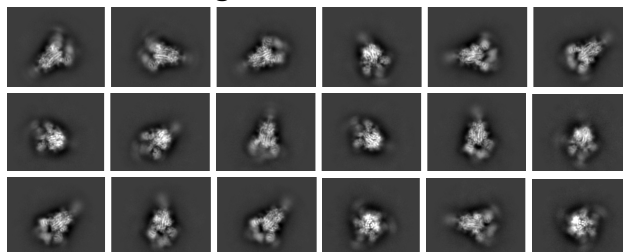

Supplement: S4 Fig — The top panel shows a representative cryo-electron micrograph of the SARS-CoV spike complexed with CR3022 Fab, whereas the bottom panels show the 2D class averages. (PDF) [file ppat.1009089.s004.pdf]

**Figure S5**

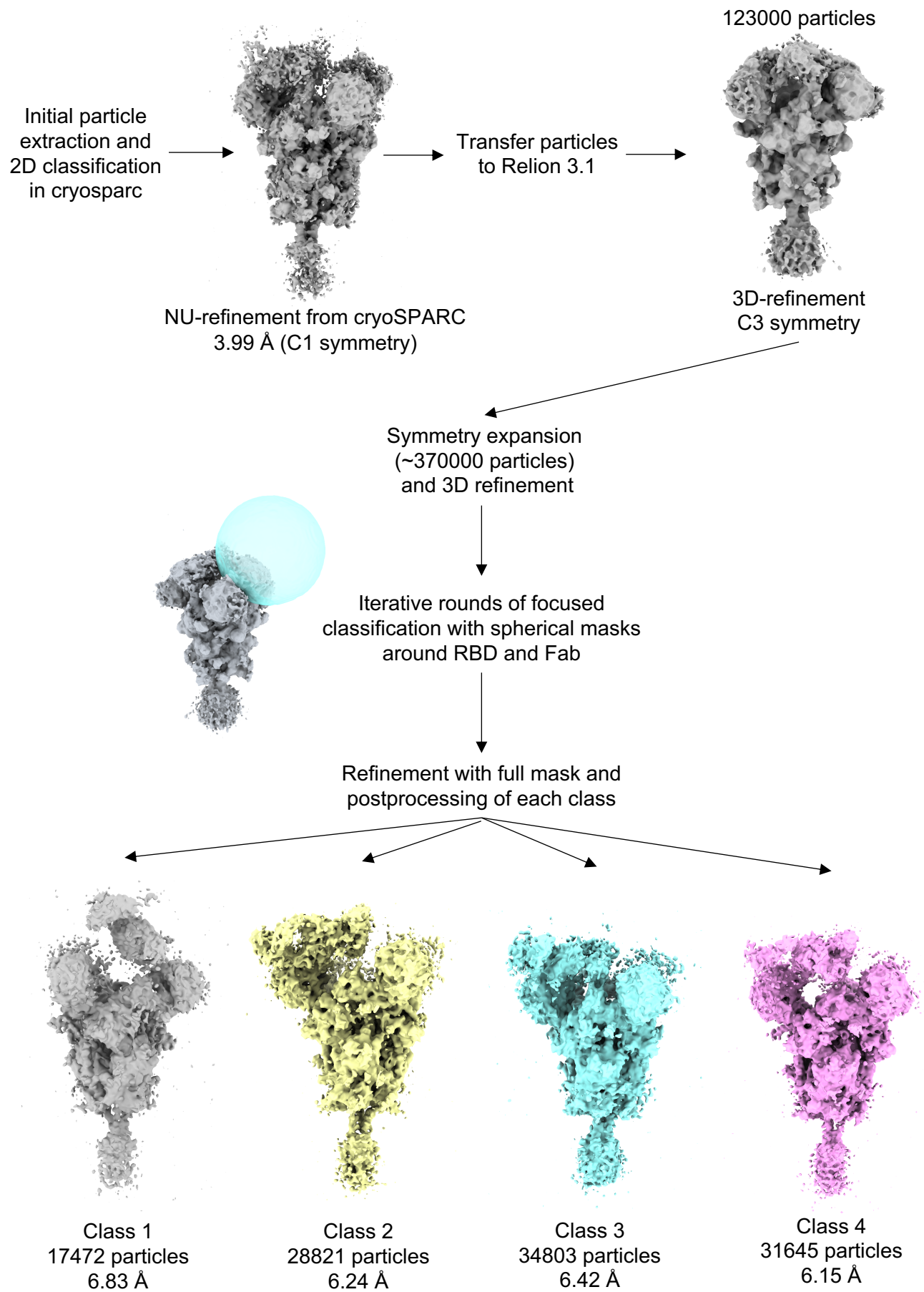

Supplement: S5 Fig — Four 3D class averages of complex of the SARS-CoV spike and CR3022 were found during data processing. (PDF) [file ppat.1009089.s005.pdf]

Figure S6

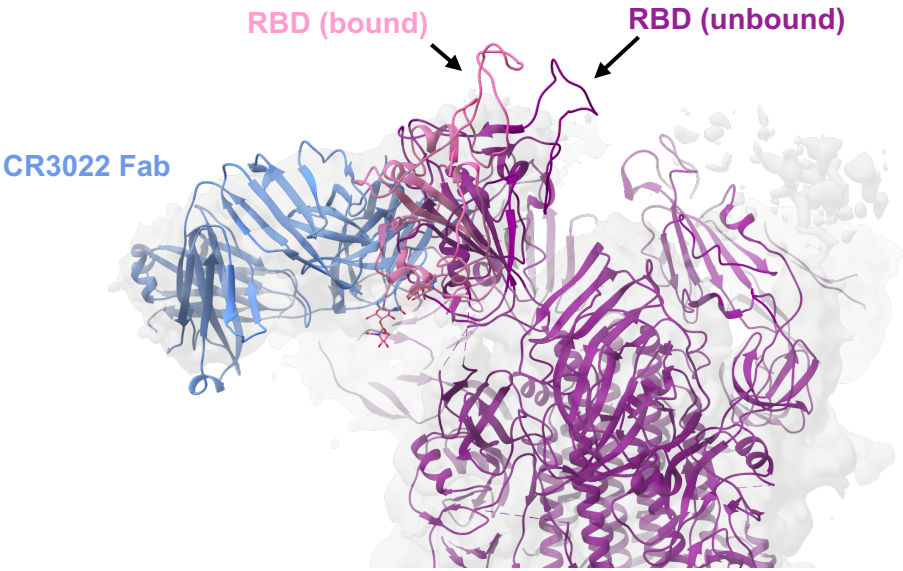

Supplement: S6 Fig — The conformation of CR3022-bound RBD in class 2 and 4 is compared to the conformation of RBD on an unliganded SARS-CoV S protein (PDB 6ACD) [35]. (PDF) [file ppat.1009089.s006.pdf]

Figure S7

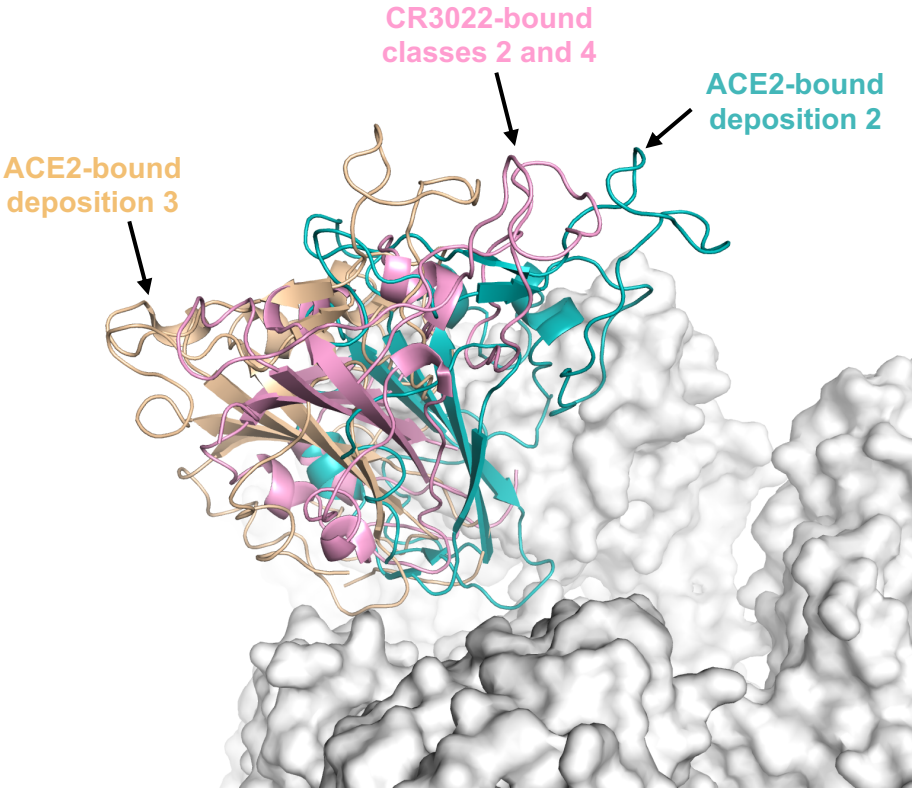

Supplement: S7 Fig — The conformation of CR3022-bound RBD in class 2 and 4 is compared to that of dispositions 2 and 3 of ACE2-bound RBD (PDB 6ACJ and 6ACK, respectively) [35]. (PDF) [file ppat.1009089.s007.pdf]

**Figure S8**

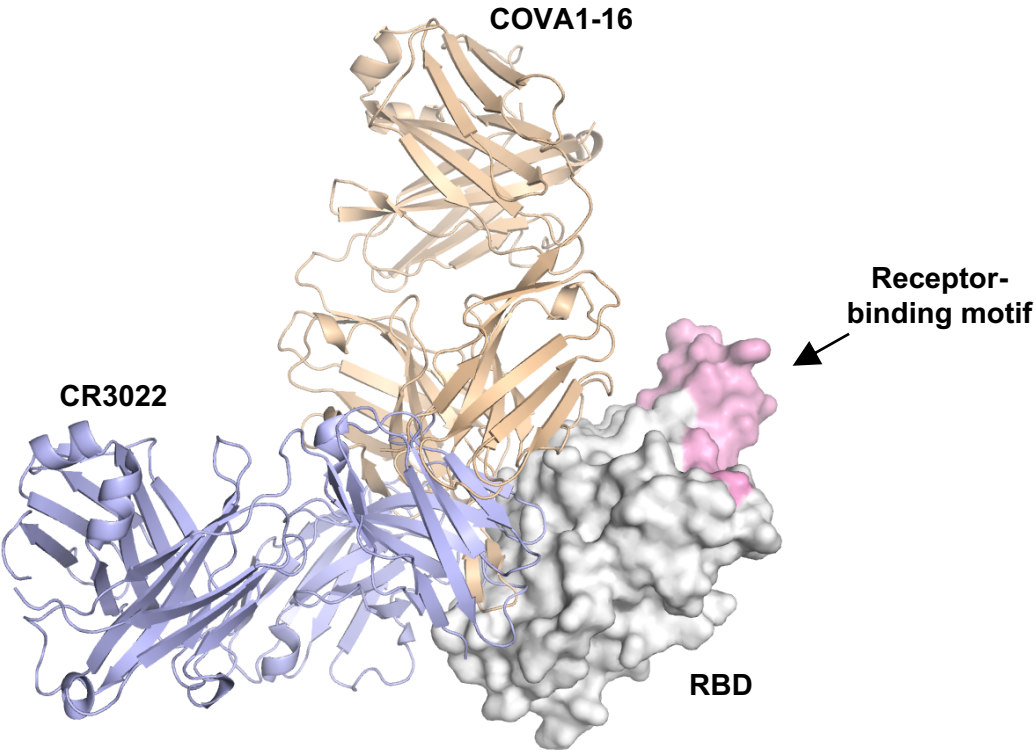

Supplement: S8 Fig — The angles of approach of CR3022 (blue) and COVA1-16 (wheat, PDB 7JMW) [39] to RBD are compared. Receptor-binding motif (residues 472–498) on the RBD is colored in pink. (PDF) [file ppat.1009089.s008.pdf]
