# Supplementary material for: A natural mutation between SARS-CoV-2 and SARS-CoV determines neutralization by a cross-reactive antibody
Source: PLoS Pathog. 2020 Dec 4;16(12):e1009089. doi: 10.1371/journal.ppat.1009089 (PMC7744049; doi:10.1371/journal.ppat.1009089)
Supplement: S1 Table — (PDF) [file ppat.1009089.s009.pdf]

Table S1

**Data collection**	
Beamline	SSRL 12-2
Wavelength (Å)	0.97946
Space group	C 1 2 1
Unit cell parameters (Å and °)	a=265.7, b=59.9, c=51.7, β=99.8
Resolution (Å)	50.0–2.70 (2.76–2.70)[a]
Unique reflections	21,547 (2,021)[a]
Redundancy	6.7 (5.5)[a]
Completeness (%)	100.0 (100.0)[a]
$<I/\sigma_I>$	14.7 (1.0)[a]
R_{sym}[b] (%)	9.2 (86.1)[a]
R_{pim}[b] (%)	5.4 (54.8)[a]
$CC_{1/2}$[c] (%)	99.4 (74.4)[a]
**Refinement statistics**	
Resolution (Å)	45.0–2.70
Reflections (work)	21,501
Reflections (test)	1,011
R_{cryst}[d] / R_{free}[e] (%)	22.2 / 27.6
No. of atoms	4,872
Macromolecules	4,795
Glycans	42
Solvent	30
Average *B*-value ($Å^2$)	80
Macromolecules	80
RBD	104
Fab	70
Glycans	30
Solvent	60
Wilson *B*-value ($Å^2$)	64
**RMSD from ideal geometry**	
Bond length (Å)	0.005
Bond angle (°)	1.17
**Ramachandran statistics (%)**	
Favored	95.6
Outliers	0.16
**PDB code**	7JN5

[a] Numbers in parentheses refer to the highest resolution shell.

[b] $R_{sym} = \Sigma_{hkl} \Sigma_i | I_{hkl,i} - <I_{hkl}> | / \Sigma_{hkl} \Sigma_i I_{hkl,i}$ and $R_{pim} = \Sigma_{hkl} (1/(n-1))^{1/2} \Sigma_i | I_{hkl,i} - <I_{hkl}> | / \Sigma_{hkl} \Sigma_i I_{hkl,i}$, where $I_{hkl,i}$ is the scaled intensity of the ith measurement of reflection h, k, l, $<I_{hkl}>$ is the average intensity for that reflection, and *n* is the redundancy.

[c] $CC_{1/2}$ = Pearson correlation coefficient between two random half datasets.

[d] $R_{cryst} = \Sigma_{hkl} | F_o - F_c | / \Sigma_{hkl} | F_o |$ x 100, where F_o and F_c are the observed and calculated structure factors, respectively.

[e] R_{free} was calculated as for R_{cryst}, but on a test set comprising 5% of the data excluded from refinement.
