# Supplementary material for: A natural mutation between SARS-CoV-2 and SARS-CoV determines neutralization by a cross-reactive antibody
Source: PLoS Pathog. 2020 Dec 4;16(12):e1009089. doi: 10.1371/journal.ppat.1009089 (PMC7744049; doi:10.1371/journal.ppat.1009089)
Supplement: S2 Table — (PDF) [file ppat.1009089.s010.pdf]

Table S2

Map	SARS-CR3022Fab Class 1	SARS-CR3022Fab Class 2	SARS-CR3022Fab Class 3	SARS-CR3022Fab Class 4
EMDB	EMD-22861	EMD-22862	EMD-22863	EMD-22864
**Data collection**				
Microscope	FEI Talos Arctica	FEI Talos Arctica	FEI Talos Arctica	FEI Talos Arctica
Voltage (kV)	200	200	200	200
Detector	Gatan K2 Summit	Gatan K2 Summit	Gatan K2 Summit	Gatan K2 Summit
Recording mode	Counting	Counting	Counting	Counting
Nominal magnification	36,000	36,000	36,000	36,000
Movie micrograph pixelsize (Å)	1.15	1.15	1.15	1.15
Dose rate (e^-/[(camera pixel)*s])	5.6	5.6	5.6	5.6
Number of frames per movie micrograph	47	47	47	47
Frame exposure time (ms)	250	250	250	250
Movie micrograph exposure time (s)	11.7	11.7	11.7	11.7
Total dose (e^-/Å^2)	50	50	50	50
Defocus range (µm)	-0.4 to -1.6	-0.4 to -1.6	-0.4 to -1.6	-0.4 to -1.6
**EM data processing**				
Number of movie micrographs	2952	2952	2952	2952
Number of molecular projection images in map	17,472	28,821	34,803	31,645
Symmetry	C1	C1	C1	C1
Map resolution (FSC 0.143; Å)	6.83	6.24	6.42	6.15
Map sharpening B-factor (Å^2)	-164.6	-147.4	-120.5	-138.1
